# Identification of rumen microbial biomarkers linked to methane emission in Holstein dairy cows

**DOI:** 10.1111/jbg.12427

**Published:** 2019-08-16

**Authors:** Yuliaxis Ramayo‐Caldas, Laura Zingaretti, Milka Popova, Jordi Estellé, Aurelien Bernard, Nicolas Pons, Pau Bellot, Núria Mach, Andrea Rau, Hugo Roume, Miguel Perez‐Enciso, Philippe Faverdin, Nadège Edouard, Dusko Ehrlich, Diego P. Morgavi, Gilles Renand

**Affiliations:** ^1^ UMR 1313 GABI INRA, AgroParisTech, Université Paris‐Saclay Jouy‐en‐Josas France; ^2^ Animal Breeding and Genetics Program IRTA Torre Marimon Caldes de Montbui Spain; ^3^ Department of Animal Genetics, CRAG UAB Bellaterra Spain; ^4^ VetAgro Sup, UMR 1213 Herbivores INRA, Université Clermont Auvergne Saint‐Genès‐Champanelle France; ^5^ INRA METAGENOPOLIS Unit Jouy‐en‐Josas France; ^6^ UMR 1348 PEGASE INRA, Agrocampus‐Ouest Saint‐Gilles France

**Keywords:** metagenomics, metataxonomics, methane emission, microbial biomarker

## Abstract

Mitigation of greenhouse gas emissions is relevant for reducing the environmental impact of ruminant production. In this study, the rumen microbiome from Holstein cows was characterized through a combination of 16S rRNA gene and shotgun metagenomic sequencing. Methane production (CH_4_) and dry matter intake (DMI) were individually measured over 4–6 weeks to calculate the CH_4_ yield (CH_4_y = CH_4_/DMI) per cow. We implemented a combination of clustering, multivariate and mixed model analyses to identify a set of operational taxonomic unit (OTU) jointly associated with CH_4_y and the structure of ruminal microbial communities. Three ruminotype clusters (R1, R2 and R3) were identified, and R2 was associated with higher CH_4_y. The taxonomic composition on R2 had lower abundance of Succinivibrionaceae and *Methanosphaera*, and higher abundance of Ruminococcaceae, Christensenellaceae and Lachnospiraceae. Metagenomic data confirmed the lower abundance of Succinivibrionaceae and *Methanosphaera* in R2 and identified genera (*Fibrobacter* and *unclassified Bacteroidales*) not highlighted by metataxonomic analysis. In addition, the functional metagenomic analysis revealed that samples classified in cluster R2 were overrepresented by genes coding for KEGG modules associated with methanogenesis, including a significant relative abundance of the methyl‐coenzyme M reductase enzyme. Based on the cluster assignment, we applied a sparse partial least‐squares discriminant analysis at the taxonomic and functional levels. In addition, we implemented a sPLS regression model using the phenotypic variation of CH_4_y. By combining these two approaches, we identified 86 discriminant bacterial OTUs, notably including families linked to CH_4_ emission such as Succinivibrionaceae, Ruminococcaceae, Christensenellaceae, Lachnospiraceae and Rikenellaceae. These selected OTUs explained 24% of the CH_4_y phenotypic variance, whereas the host genome contribution was ~14%. In summary, we identified rumen microbial biomarkers associated with the methane production of dairy cows; these biomarkers could be used for targeted methane‐reduction selection programmes in the dairy cattle industry provided they are heritable.

## 1 INTRODUCTION

Cattle have the remarkable ability to digest and transform non‐edible plant cell wall components into high‐quality proteins for human consumption. The digestion of feeds entails, however, the production of the greenhouse gas CH_4_. The possibility to mitigate CH_4_ emissions while simultaneously improving feed efficiency is highly relevant for the sustainability of cattle production systems. Notwithstanding, the mechanisms to achieve it are not yet fully understood (Flay et al., 2019). The contribution of the gastrointestinal microbiota to feed digestion and enteric CH_4_ production is well established in ruminants (Delgado et al., 2019; Difford et al., 2018; Huws et al., 2018; Ross, Moate, Marett, Cocks, & Hayes, 2013; Tapio, Snelling, Strozzi, & Wallace, 2017). However, enteric CH_4_ production is a complex trait determined not only by the rumen microbiome (Huws et al., 2018; Ross et al., 2013; Tapio et al., 2017), but also by host genetics (Difford et al., 2018; Ross et al., 2013), and environmental factors (Gerber et al., 2013; Martin, Morgavi, & Doreau, 2009; McAllister, Cheng, Okine, & Mathison, 1996). Exploring the abundance and composition of microbial communities in the gastrointestinal tract of cattle in relation to the host genome is of great interest for quantifying animal variability in feed digestibility and enteric CH_4_ emission (Huws et al., 2018; Leahy et al., 2013; Li et al., 2019). Several studies in ruminants have explored the relationship of the rumen microbiota composition with feed efficiency and CH_4_ emission (Delgado et al., 2019; Jami, White, & Mizrahi, 2014; Jewell, McCormick, Odt, Weimer, & Suen, 2015; Li et al., 2019; Myer, Smith, Wells, Kuehn, & Freetly, 2015; Wallace et al., 2015). Pioneering rumen‐engineering studies have suggested that microbial communities are highly resilient and host‐specific (Cole, 1991; Weimer, 2015). Although these latter properties make it difficult to manipulate the ruminal microbial community, they also enable the analysis of the covariation of these ecosystems with host performance and jointly selecting both host genome and microbiome variants.

The main goal of this study was to implement an integrative approach using 16S rRNA and shotgun metagenomic sequencing data to identify microbial biomarkers linked to CH_4_y emission. In addition, we also explored the role of host genetics on the determinism of this phenotype.

## 2 MATERIALS AND METHODS

### 2.1 Phenotype and host genotype details

The experiment was carried out at the INRA experimental farm in Méjusseaume (Le Rheu, France). Management of experimental animals followed the guidelines for animal research of the French Ministry of Agriculture and other applicable guidelines and regulations for animal experimentation in the European Union (European Commission, 2010). Approval number for ethical evaluation was APAFIS:3122‐2015112718172611. Sixty‐five loose‐housed lactating Holstein cows were used in this experiment. They were allocated to three pens equipped with individual troughs and automatic gates detected by radio‐frequency identification tags attached on the cow ears. Each pen was of similar size (*n* = 21–23). Parity was equilibrated between pens, with two‐thirds of the cows that were in their first lactation (*n* = 42). Cows received the same total mixed ration (TMR) throughout the experiment consisting of maize silage (65%), soybean cake (18%) and energy concentrate (8%), composed of corn, wheat, barley, dehydrated beet pulp, dehydrated alfalfa (8%) and minerals (Table S1). The TMR was offered ad libitum and individually weighed every morning. Each morning, refusals from the previous day were weighed. Samples of forages, concentrates and refusals were analysed for dry matter content. Dry matter intake was calculated daily as the difference between offered and refused dry matter weights.

Methane production was measured with two GreenFeed emission monitoring (GEM) systems (C‐Lock Inc.). This system automatically measures CH_4_ when animals visit a concentrate feeder equipped with a head hood and an extractor fan for the capture of breath and eructation gases. The animals are attracted and kept attracted to the feeder with pellets that are distributed in small quantities. In the current experiment, the cows were allowed to visit the GEM a maximum of four times per day, with each visit separated by at least 6 hr. At each visit, cows received six drops of concentrate separated by 30 s for at least 3 min. At each visit, CH_4_ production rate is calculated, combining the gas concentration (measured with a non‐dispersive infrared analyser) to the airflow in the pipe (measured with a flow meter). An algorithm developed and applied by C‐Lock Inc. calculates the CH_4_ rate at each visit if the head of the animal is correctly positioned (controlled by a laser beam) for at least 2 min. With two GEM systems and three batches, the following experimental design was applied: during a first period (6 weeks in January–February 2017), cows in batches B1 (*n* = 21) and B2 (*n* = 21) were measured and rumen liquid sampled. During a second period (the next 4 weeks in March 2017), cows in batch B1 were measured again and sampled together with cows in batch B3 (*n* = 23). The daily dry matter intake (TMR and concentrate in the GEM system) and the per‐visit CH_4_ emission rate measures were averaged over the testing periods to estimate the individual dry matter intake (DMI), methane emission rate (CH_4_) and methane yield (CH_4_y = CH_4_/DMI). The traits were adjusted beforehand for the contemporary group mean in a simple linear model including the batch × period effect:

y=Xb+e

where *y* is the trait value vector, *b* the fixed effect vector of the contemporary group (batch × period) effect and *e* the residual phenotype.

Rumen fluid (~400 ml) was sampled via oesophageal tubing in the morning before feeding (last week of February for period 1 and last week of March for period 2). The average days in milk (DIM) in period 1 were 148.5 (*SD* 12.5) and 182.8 (*SD* 18.6) in period 2. Samples were filtered through a polyester monofilament fabric (250 μm mesh aperture), and 2 ml of the filtrate was centrifuged at 20,000 *g*, 20 min, 4°C. The supernatant was discarded, and the pellet was snap‐frozen in liquid nitrogen and stored at −80°C.

The Illumina BovineSNP50 v.2 BeadChip (Illumina Inc.) was used to genotype the 65 Holstein cows. Quality control was performed to exclude single nucleotide polymorphisms (SNPs) with minor allele frequencies (MAF) <5%, rates of missing genotypes above 10%, as well as those that did not conform to Hardy–Weinberg expectations (threshold set at a *p*‐value of .001). We also excluded SNPs that did not map to the bovine reference genome (ARS‐UCD1.2 assembly) or that were located on the X‐chromosome.

### 2.2 Rumen microbial DNA extraction, PCR amplification and sequencing

DNA from rumen liquid fraction was extracted with an established protocol (Yu & Morrison, 2004). Extracted DNA was sent to the University of Illinois Keck Center for Fluidigm sample preparation and Illumina sequencing. Primers targeting the V3–V5 region (F357 and R926) were used to amplify a region of 570 base pairs of the bacterial 16S rRNA gene. Archaea‐specific primers (349F and 806R) were used to amplify a 457‐base‐pair 16S rRNA gene fragment. The amplicons were sequenced on one MiSeq flow cell for 251 cycles. The whole‐metagenome shotgun sequence of 30 samples collected from rumen of Holstein cows with low and high CH_4_y emission and distributed across the three batches were generated using a quantitative metagenomic pipeline (Supporting Information).

### 2.3 Bioinformatics and statistical analysis

Sequences corresponding to the 16S rRNA gene data were analysed on an in‐house Galaxy‐based graphical user interface for IM TORNADO (Jeraldo et al., 2014) and mothur (Schloss et al., 2009) for bacteria and archaea, respectively. The workflow included a quality control step to remove sequences with Phred scores of <33 and trimmed sequences based on expected amplicon length, as well as merge paired reads, remove chimera and select OTUs (97% identity). Finally, after removing doubleton OTUs, only those OTUs representing more than 0.001% of the total were retained. Taxonomic classification was based on the SILVA v123 database (Quast et al., 2012) for bacteria and RIMDB (Seedorf, Kittelmann, Henderson, & Janssen, 2014) for archaea. As previously mentioned, animals in the study were divided into groups due to experimental constraints (see Phenotype2.1 section). Therefore, to estimate the stability of ruminal bacterial communities, we used the 16S rRNA gene data of 21 cows in batch B1 that were sampled twice in the two successive periods. In a first step, we estimated and contrasted diversity metrics among time points (T1 vs. T2) with vegan r package (Jari Oksanen et al., 2018). Alpha‐diversity was evaluated with the Shannon index (Shannon, 1984), and beta‐diversity was assessed using the Whittaker index (Whittaker, 1972). Subsequently, stability between sampling points was estimated using the RV coefficient on the two OTU normalized abundance tables. The RV coefficient was calculated between times points as the total co‐inertia (sum of eigenvalues of the product of two cross‐product matrices) divided by the square root of the product of the squared total inertia. A zero RV score indicates no similarity, whereas the RV score approaches 1 for increasing co‐structure between two data sets.

The whole‐metagenome data were processed as follows. Gene abundance profiling was performed using the 16.6 million gene integrated reference catalogue of the rumen microbiome (Junhua et al., 2019). First, low‐quality and host contaminant reads (from Bos taurus genome ARS‐UCD1.2) were removed using AlienTrimmer (Schubert, Lindgreen, & Orlando, 2016) and Bowtie2 (Langmead & Salzberg, 2012), respectively. For a more detailed information which includes the creation of gene abundance and KEGG orthologous (KO) tables, as well as the assembly of metagenomic species (MGS) clusters, see the supplementary material (Supporting Information).

#### 2.3.1 Structure of the ruminal ecosystem

To infer the structure of ruminal bacterial communities, ruminotype cluster detection was done using the genera abundance (101 genera based on the bacterial data) in each sample, as previously described for human gut enterotypes (Arumugam et al., 2011). Briefly, sample clusters were detected using the probability distribution distance metric related to Jensen–Shannon divergence and partitioning around medoids. The optimal number of clusters was determined following the Calinski–Harabasz (CH) index (Caliński & Harabasz, 1974), and the statistical consistency of the corresponding partition was evaluated using the Silhouette coefficient (Rousseeuw, 1987). Furthermore, sample stability within each cluster was estimated through a cluster‐wise Jaccard bootstrap analysis (100 repetitions; Hennig, 2007). The association between predicted ruminotype clusters with CH_4_y was obtained using a least‐squares analysis as implemented in the lsmeans r package. To identify genera with significantly different abundance among the predicted ruminotype cluster groups, a differential abundance (DA) analysis was performed using the zero‐inflated Gaussian mixture model implemented in the metagenomeseq r package (Paulson, Stine, Bravo, & Pop, 2013), using a threshold for adjusted *p*‐values of 5%. In addition, a presence–absence (PA) test was also performed to identify genera that were unique for each of the identified clusters.

#### 2.3.2 Multivariate analysis

To identify a set of OTUs jointly associated with CH_4_y phenotypic variation and the structure of ruminal bacterial communities, a combination of multivariate analyses was performed using sparse partial least‐squares (sPLS) as implemented in the mixomics r package (Rohart, Gautier, Singh, & Lê Cao, 2017). sPLS is a statistical approach employed to identify a small subset of variables that maximize the covariance between two different data sets (for instance, a table of centred log ratio‐transformed OTUs and ruminotype clusters or CH_4_y values; Lê Cao, González, & Déjean, 2009). In a first step, sPLS discriminant analysis (sPLS‐DA) was applied based on sample classification according to ruminotype cluster assignment. The classification reliability corresponding to the sPLS‐DA model was assessed as function of the prediction maximum distance between overall misclassification error rate and balanced error rate (BER) after fivefold cross‐validation repeated 500 times. In addition, sPLS was also performed in regression model to identify OTUs associated with CH_4_y phenotypic variation. Subsequently, we implemented a conservative approach and retained only those OTUs found in common between the two approaches for downstream analysis. The sPLS‐DA approach using ruminotype‐like sample classification was also implemented on the metagenomic data at MGS and functional (KEGG) levels.

#### 2.3.3 Mixed model

To estimate the proportion of CH_4_ and the CH_4_y phenotypic variance explained by the host (heritability) as well as by the bacterial community (microbiability), the following Bayesian mixed model was implemented using the bglr r package (Pérez & de los Campos, 2014):

y=1μ+g+b+ε

where ***y*** is the phenotype vector (i.e., CH_4_y), **1*µ*** is the intercept, $g∼N\left(0,{G\sigma }_{g}^{2}\right)$ and $b∼N\left(0,{B\sigma }_{b}^{2}\right)$, ***G*** is the genomic relationship matrix (GRM) based on 38,872 autosomal SNPs, and $\sigma_{g}^{2}$ represents the additive genetic variance. ***B*** represents the microbial relationship matrix, calculated based on the Bray–Curtis dissimilarities distance matrix, and ***ε*** is the residual term. A second model was employed using the method proposed by Ross et al. (2013) which build the microbial relationship matrix based on the variance–covariance matrix from the log‐transformed and standardized OTU table. In both cases, the models were run using a Gibbs sampler with 30,000 iterations and a burn‐in of 2,000 rounds; we used standard flat priors for the intercept.

## 3 RESULTS AND DISCUSSION

### 3.1 Phenotype description

The descriptive statistics of production and CH_4_ traits are presented in Table 1. Methane emissions were calculated as the average value of all visits throughout the study. With 2.50 visits per day, emission rates per each cow were calculated for an average of 105.1 and 69.7 visits for periods 1 and 2, respectively. This large number of visits to GEM system ensured a precise measurement of the animal phenotype, as it largely exceeds the recommended 20–30 minimum visits (Barchia et al., 2017; Manafiazar, Zimmerman, & Basarab, 2016). The variability among cows for methane production (g/day; CV 11%) was higher than previously reported (Ricci, Rooke, Nevison, & Waterhouse, 2013). From period 1 to period 2, the repeatability of the methane emission traits was 0.82 and 0.73 for CH_4_ and CH_4_y, respectively. These values were lower than the repeatability of production traits (0.95–0.97 for DMI, milk production and milk efficiency). Methane production rate was moderately correlated with DMI (Pearson's correlation *r* = .44) and milk production (*r* = .28) and was poorly correlated with body weight (*r* = .16).

**Table 1:** Mean and standard deviation of production and methane emission traits

Trait	Unit	Mean	*SD*	CV (%)
Live weight	kg	634	49	8
Dry matter intake (DMI)	kg/day	21.2	2.2	10
Milk production	kg/day	31.1	4.8	15
Milk efficiency	kg/kg DMI	1.47	0.16	11
GreenFeed visits	*n*/day	2.50	0.53	21
Visit duration	s	224	16	7
Methane emission rate	g/day	506	56	11
Methane yield	g/kg DMI	24.1	3.1	13

### 3.2 Link between ruminotype structure of ruminal bacterial communities and CH_4_y emissions

**Figure 1:**
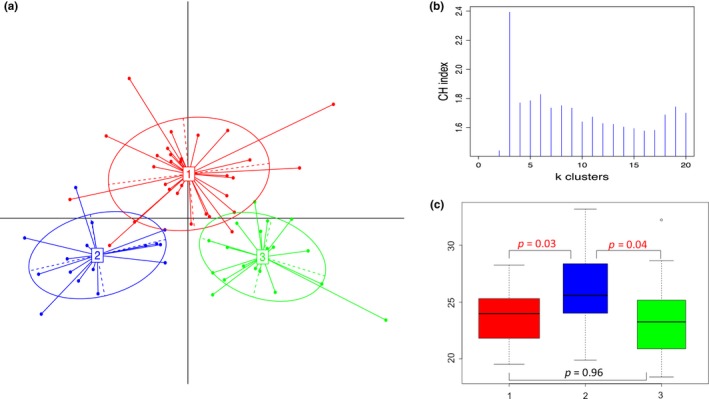
Structure of the ruminal bacterial community of lactating Holstein cows displaying natural differences in methane emission. (a) Sample distribution. (b) Calinski–Harabasz (CH) indexes of potential numbers of clusters. (c) Pairwise comparisons of CH_4_y emission between ruminotype‐like clusters R1 (red), R2 (blue) and R3 (green)

The 16S rRNA gene sequences from 65 rumen liquid fractions were analysed. After quality control, bacterial 1,198 OTUs and 1,764 archaeal OTUs were identified (Supporting Information). The relative abundance of bacterial genera in each sample was used for cluster detection as described for human gut enterotypes (Arumugam et al., 2011). This method first performs a sample stratification, followed by the identification of the optimal number of clusters and the statistical consistency of the predicted partition. Cows clustered into three ruminotype clusters (R1, R2 and R3; Figure 1a,b); 30 cows were assigned to R1, 16 to R2 and 19 to R3. In concordance with our results, three ruminotype clusters have previously been reported in sheep (Kittelmann et al., 2014), while in dairy cattle (based on a principal coordinate analysis at the OTU level), two clusters were reported by Danielsson et al. (2017). Therefore, we tested sample stability by comparing two (*k* = 2) or three (*k* = 3) putative clusters. Cluster‐wise Jaccard bootstrap analysis revealed moderate sample stability, showing a more stable solution for three (K3: 0.63, 0.64 and 0.63) clusters compared with two (K2: 0.61 and 0.4). In concordance with the aforementioned studies in sheep (Kittelmann et al., 2014) and dairy cattle (Danielsson et al., 2017), we observed a significant association between ruminotype cluster assignments and CH_4_y emission (Figure 1c). Cows that clustered within R2 emitted more CH_4_y (*p* < .05) than those clustered in R1 or R3, and no significant difference in CH_4_y emission was observed between the R1 and R3 clusters (Figure 1c).

We evaluated the taxonomic composition of ruminotype clusters through a combination of presence–absence (PA) and differential abundance (DA) analysis. According to the PA test, the only genera showing significant differences between clusters was *Succinivibrionaceae_UCG‐001*, which was absent in most cows classified as R2 (high CH_4_y emission) compared with R1 and R3. Only four cows out of the 16 in R2 had *Succinivibrionaceae_UCG‐001*, albeit with a lower abundance compared to the other clusters (Table 2). The DA analysis confirmed the PA test results, as *Succinivibrionaceae_UCG‐001* showed significantly different abundance among clusters (Table S2). We note that larger differences in *Succinivibrionaceae_UCG‐001* abundance, as well as a greater number of DA genera, were observed between samples classified as R2 and R3 (Table S2). However, no significant difference in *Succinivibrionaceae_UCG‐001* relative abundance was observed between R1 and R3 (Table S2). These observations are consistent with previous reports describing members of the Succinivibrionaceae family associated with low CH_4_ (Danielsson et al., 2017; Kittelmann et al., 2014; Wallace et al., 2015). Furthermore, this is in agreement with the higher CH_4_y emissions observed in R2. Members of families associated with high CH_4_ emission, such as Ruminococcaceae, Christensenellaceae and Lachnospiraceae (Kittelmann et al., 2014; Tapio et al., 2017), exhibited DA patterns with higher abundant in R2 compared to R1 and R3 (Table S2). In a similar way, we explored the archaeal taxonomic composition of the ruminotype clusters. According to a DA analysis, significant differences were only detected at the OTU level. Similar to the aforementioned patterns at the bacterial level, the largest number of DA OTUs was observed between samples classified as R2 and R3 (Table S3). Of noted, OTU members of *Methanosphaera* showed consistently lower abundance in the R2 cluster (higher CH_4_ emitters) and were only detected when R2 was compared with R1 or R3. Similar patterns were observed for OTUs classified as *Methanobrevibacter ruminantium*, which showed lower abundance in R2 compared with R3. Meanwhile, no clear patterns were observed for OTU members of the genera Methanobrevibacter (*gottschalkii* clade); some OTUs were most abundant in R2, while others showed higher abundance in R1 or R3 (Table S3).

**Table 2:** Results from the presence–absence test between ruminotype‐like clusters

Comparison	Genus	Odds ratio	*p* Values	Adj *p* values (BH)
R1_R2	Succinivibrionaceae_UCG‐001	17.72	5.27E−05	.005
R2_R3	Succinivibrionaceae_UCG‐001	0.022	4.22E−05	.003

### 3.3 Identification of ruminal OTUs linked to CH_4_y emission and associated with the structure of the rumen microbiota

**Figure 2:**
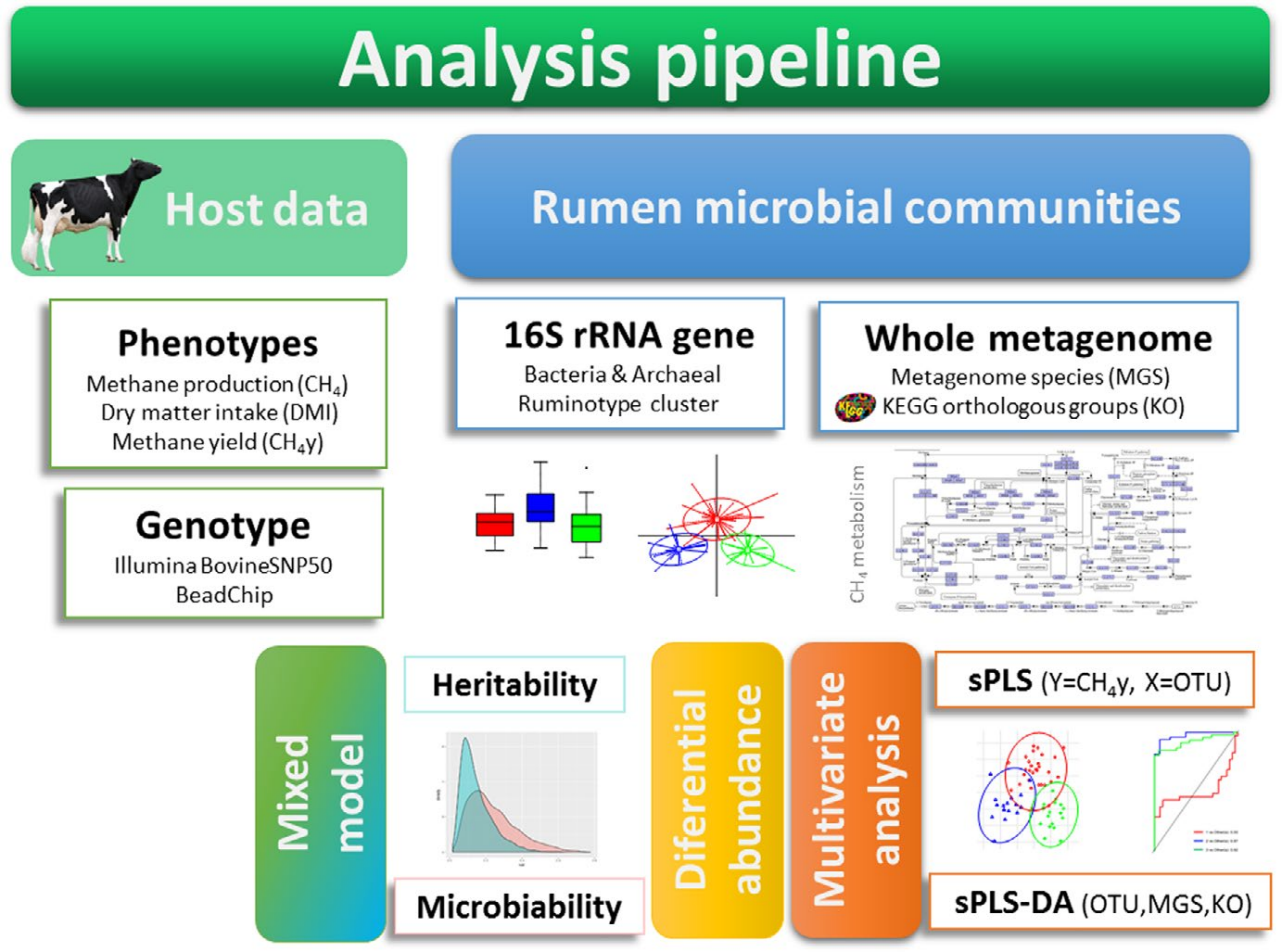
Analytical framework employed to identify microbial biomarkers of methane emission in lactating Holstein cows

In this study, we used an integrative approach combining multivariate and clustering analyses to identify microbial biomarkers linked to CH_4_y emission and the structure of the rumen microbiota (Figure 2). We are aware that in comparison with a predictive model (which would take into account the total microbial variation), ruminotype cluster approaches may have some limitations for biomarker identification (Costea et al., 2018; Knights et al., 2014). However, as previously reported (Danielsson et al., 2017; Kittelmann et al., 2014) and confirmed in this work, the link between the structure of the bacterial ruminal ecosystem and CH_4_y emission cannot be neglected. Therefore, to conservatively focus on the primary OTU markers associated with CH_4_y emissions, we combined the cluster analysis results with those of a predictive model. We applied a two‐pronged strategy using multivariate analysis, including a supervised sPLS‐DA (based on sample cluster classification) and a sPLS regression model that considers the joint covariation of OTU relative abundances and CH_4_y emissions (Figure 2). For the sPLS‐DA, the first and second principal component combined the relative abundances of 231 OTUs (PC1 = 200 and PC2 = 31) and allowed a clear discrimination between samples classified as R2 and R3 (Figure S2 and Table S4). The area under the ROC curve corresponding to both R2 (0.97) and R3 (0.92) showed high values, suggesting a good ability of the model to correctly classify these samples. In addition, the sPLS regression model allowed us to identify features that maximize the covariance between OTU relative abundance and CH_4_y phenotype variation. After tuning parameters (fivefold cross‐validation repeated 500 times), a single component was identified as optimal (PC1 $Q_{h}^{2}$ = 0.12), yielding a final selection of 200 OTUs. We then applied a conservative approach, retaining the 86 OTUs that were common to both sPLS analyses (Table S4). We note that 75.5% (65/86) of them were also identified as DA among ruminotype clusters, where selected OTUs displayed consistent differential abundance patterns (Table S4). The taxonomic classification of these OTUs included families reportedly linked to CH_4_ such as Succinivibrionaceae, Ruminococcaceae, Christensenellaceae, Lachnospiraceae, Gastranaerophilales, Rikenellaceae, Bacteroidales_BS11 and Prevotellaceae (Danielsson et al., 2017; Difford et al., 2018; Kamke et al., 2016; Kittelmann et al., 2014).

### 3.4 Rumen microbiome linked to CH_4_y identified through metagenomics

To gain insight into a more precise taxonomic composition and functional processes that differed between ruminotypes, we generated whole‐metagenome data from 30 samples distributed across the two periods and the three ruminotype clusters. After removing low‐quality and host contaminant reads (Supporting Information), an average of 21.3 million reads per sample was produced. Reads were mapped against the rumen gene reference catalogue (Junhua et al., 2019). A total of 608 MGS were identified by MSPminer (Cervino et al., 2019) and 264 KEGG functional modules were detected by consolidating the gene abundance matrix into KEGG annotations (Supporting Information). In a first step, the overall structure of the ruminal microbial community was explored using non‐metric multidimensional scaling (nMDS) with Bray–Curtis and binary Jaccard dissimilarities inferred from the metagenomic gene abundance matrix (Figure S3). nMDS plots showed a clear relationship between ruminotypes and nMDS patterns for both dissimilarities. After correction for group and period effects, PERMANOVA tests of significance also showed a significant association between ruminotype classification and Bray–Curtis (*p* < .05) and binary Jaccard (*p* < .01) dissimilarity matrices. The same approach was followed to explore the relationship between rumen ecosystem and CH_4_y, with nMDS plots showing a tendency of samples to group according to CH_4_y values. In this case, the PERMANOVA test corrected by group and period was not significant for Bray–Curtis or binary Jaccard (*p* = .06) dissimilarities.

The MGS and functional profiles of each sample were explored through sPLS‐DA. Based on MGS, the relative abundance of 150 MGS enabled the discrimination between samples (Table S5 and Figure S4). In agreement with the taxonomic classification of the 86 selected OTUs, members of the Succinivibrionaceae, Lachnospiraceae, Prevotellaceae, Rikenellaceae and Ruminococcaceae families were among the top discriminant MGS. Moreover, 59.3% (89/150) of the MGS were also identified as DA among ruminotype (Table S6). Similarly, to the aforementioned results at the OTU level, a larger number of DA MGS were observed between samples classified as R2 and R3 (Table S6). Interestingly, shotgun metagenomics shows a lower abundance of a MGS affiliated to Succinivibrionaceae (MGS116, fold change_R3,R2_ = 1.87, corrected *p*‐value = .043) in the R2 ruminotype, a finding that agrees with the results of the 16S rRNA gene data. Members of the Succinivibrionaceae have been previously proposed to be responsible for the lower CH_4_ emissions in wallaby microbiota (Pope et al., 2011). Moreover Succinivibrionaceae was recently identify among the core of heritable rumen bacteria (Wallace et al., 2019) as well as associated with feed efficiency and levels of propionate in beef cattle (Li et al., 2019). Succinivibrionaceae produce succinate as a main fermentation product (O'Herrin & Kenealy, 1993; Santos & Thompson, 2014), which is converted to propionate by other members of the microbiota and thus less hydrogen might be available for methanogens. Co‐exclusion between Succinivibrionaceae and *Methanobrevibacter* has been reported in cattle (McCabe et al., 2015). The use of metagenome data allows identifying other features not revealed by 16S rRNA gene sequences including *unclassified Bacteroidales* and *Fibrobacteres* (Table S6) as well as to confirm the patterns of methanogenic archaea members of the family Methanobacteriaceae observed using the metataxomic approach. This is in agreement with the previously reported negative correlation between *Methanosphaera* spp. and CH_4_ production in sheep (Kittelmann et al., 2014). The only MGS taxonomy classified as *Methanosphaera* (MGS512) showed lower abundance in samples classified as R2 compared with R1 and R3 (fold change_R1,R2_ = 5.92, corrected *p*‐value = .03; fold change_R3,R2_ = 8.56, corrected *p*‐value = .008).

A functional approach using KEGG modules shows a classification of samples according to ruminotype cluster assignation. The sPLS‐DA analysis revealed 55 modules; however, the model showed a slightly lower accuracy than for OTUs and MGS analyses (Table S7 and Figure S5). Among the discriminant KEGG modules, there were functional modules related to CH_4_y emission, such as methanogenesis (M00567), C4‐dicarboxylic acid cycle, NAD‐malic enzyme type (M00171, M00172) and acetyl‐CoA pathway (M00422), but also with general functions such as glycolysis (M00001), gluconeogenesis (M00003), formaldehyde assimilation (M00345) and crassulacean acid metabolism (M00169; Table S7). A detailed examination of KOs involved in the methanogenesis pathway (M00567) revealed that six of them were significantly more abundant in samples classified as R2 (Figure S6). Interesting, five of them (K00205, K00672, K00399, K00580 and K03389) including the methyl‐coenzyme M reductase (which catalyses the rate‐limiting CH_4_ synthesis (Scheller, Goenrich, Thauer, & Jaun, 2013; Wongnate et al., 2016) have been suggested as biomarkers for CH_4_ production across diverse cattle breeds (Auffret et al., 2018). Overall, our results show that shotgun metagenomics is able to provide additional insights into the differences observed with 16S rRNA gene data sets, even with smaller sample sizes. Interestingly, specific KEGG pathways and enzymes appear to be associated with CH_4_ and had a moderate discriminant ability of whole‐metagenome data using the sPLS‐DA approach.

### 3.5 CH_4_ emission heritability and microbiability

A mixed model was implemented to estimate the proportion of CH_4_ and CH_4_y phenotypic variance explained by the host cow genome, the whole OTU matrix (*n* = 1,198) and the 86 selected OTUs (Figure 2). According to the model based on the whole OTU matrix, the host genome explained ~14% of the CH_4_ and CH_4_y, whereas the bacterial community explained around ~16% of CH_4_ production and 18% of CH_4_y (Table 3). The estimated CH_4_ heritability and microbiability on the whole OTU model were consistent with recent results in dairy cattle (Difford et al., 2018). Meanwhile, in agreement with previous studies (Saborío‐Montero, 2018), differences between the estimated microbiability were observed depending on the method employed to build the microbial relationship matrix (Table 3). We acknowledge that the number of samples in our study may not be large enough to accurately estimate parameters, which limit the robustness of the model. In spite of this limitation, consistent values of CH_4_ heritability between models were observed (Table 3). These values are also comparable to those of Difford et al. (2018). It is noted that heritability is similar for the two CH4 emission traits but microbiability was always higher when using CH_4_y. Our results also suggest a slight improvement of CH_4_y variance component estimation (up to ~24%) after OTU preselection, but no for CH_4_ alone (Table 3). Further studies with larger sample sizes and standardized analytical pipelines could provide more reliable estimates of the microbiota contribution to this complex CH_4_ emission trait.

**Table 3:** Estimated heritability (*h* ^2^) and microbiability (*m* ^2^) of methane production (CH_4_) and methane yield (CH_4_y) of lactating Holstein cows

Trait	Whole OTU table (1,198)		86 selected OTUs (Bray–Curtis distance)		86 selected OTUs (log‐transformed and standardized OTU table)^a^	
	*h* ^2^ (*SD*)	*m* ^2^ (*SD*)	*h* ^2^ (*SD*)	*m* ^2^ (*SD*)	*h* ^2^ (*SD*)	*m* ^2^ (*SD*)
CH_4_	0.144 (0.09)	0.164 (0.10)	0.141 (0.09)	0.192 (0.11)	0.157 (0.09)	0.130 (0.06)
CH_4_y	0.148 (0.10)	0.181 (0.11)	0.143 (0.09)	0.242 (0.14)	0.163 (0.09)	0.174 (0.08)

a Method proposed by Ross et al., 2013. Estimated a microbial relationship matrix based on the variance–covariance matrix from the log‐transformed and standardized OTU table.

## 4 CONCLUSION

Our results confirm the link between the structure of the ruminal bacterial community and CH_4_ emission. We identified 86 OTUs simultaneously linked to CH_4_y emission and the ruminal bacterial community structure. OTUs associated with CH_4_y emission were predominantly hydrogen‐producing bacteria and explained up to 24% of the CH_4_y phenotypic variance, whereas the host genome contribution was around 14%. Some discriminant bacterial OTUs identified by metataxonomic were confirmed by whole metagenome. In particular, samples clustered in R2 (high CH_4_y emission) showed a lower abundance of *Succinivibrionaceae* and *Methanosphaera* spp. as well as a higher abundance of genes coding for functional modules and enzymes involved in methanogenesis. Overall, we report a set of microbial biomarkers that have the potential to be employed for characterizing high‐emitting cattle for targeted management in the dairy cattle industry.

## CONFLICT OF INTEREST

The authors declare that there is no conflict of interest.

## Data Availability

The data that support the findings of this study are available from APIS‐GENE. Restrictions apply to the availability of these data, which were used under license for this study. Data are available from the corresponding author with the permission of APIS‐GENE.
