# Supplementary material for: Identification of rumen microbial biomarkers linked to methane emission in Holstein dairy cows
Source: J Anim Breed Genet. 2019 Aug 16;137(1):49–59. doi: 10.1111/jbg.12427 (PMC6972549; doi:10.1111/jbg.12427)
Supplement: Supplementary file 1 [file JBG-137-49-s001.pdf]

Ingredients (% of DM)	
Maize silage	65
Soybean meal	18
Dehydrated lucerne	8
Concentrate mix[1]	8
Mineral-vitamin mix	1
Chemical composition (% of DM)	
OM	94,9
CP	15,0
NDF	36,9
ADF	20,0
Starch	19,7
Ether Extract	2,4
Gross energy (MJ/kg of DM)	18,8

[1] Concentrate mix (%) wheat (20), maize (20), barley (20), dehydrated beet pulp (20), wheat bran (15), cane molasses (3), vegetal oil (1), salt (1)
