# Supplementary material for: Identification of rumen microbial biomarkers linked to methane emission in Holstein dairy cows
Source: J Anim Breed Genet. 2019 Aug 16;137(1):49–59. doi: 10.1111/jbg.12427 (PMC6972549; doi:10.1111/jbg.12427)
Supplement: Supplementary file 5 [file JBG-137-49-s005.pdf]

MGS	Family	Genus
MGS184	Prevotellaceae	*Prevotella*
MGS073	Prevotellaceae	*Prevotella*
MGS541	unclassified Bacteroidales	*unclassified Bacteroidales*
MGS566	Prevotellaceae	*Prevotella*
MGS339	Ruminococcaceae	*unclassified Ruminococcaceae*
MGS124	unclassified Bacteroidales	*unclassified Bacteroidales*
MGS042	unclassified Clostridiales	*unclassified Clostridiales*
MGS234	Rikenellaceae	*Alistipes*
MGS208	unclassified Bacteroidales	*unclassified Bacteroidales*
MGS207	unclassified	*unclassified*
MGS441	unclassified	*unclassified*
MGS078	Prevotellaceae	*Prevotella*
MGS136	Prevotellaceae	*Prevotella*
MGS351	Prevotellaceae	*Prevotella*
MGS025	unclassified Clostridiales	*unclassified Clostridiales*
MGS422	Prevotellaceae	*Prevotella*
MGS625	unclassified Bacteroidales	*unclassified Bacteroidales*
MGS411	unclassified Bacteroidales	*unclassified Bacteroidales*
MGS460	Prevotellaceae	*Prevotella*
MGS387	unclassified Bacteroidales	*unclassified Bacteroidales*
MGS180	Ruminococcaceae	*Ruminococcus*
MGS453	unclassified Spirochaetales	*unclassified Spirochaetales*
MGS273	unclassified Clostridiales	*unclassified Clostridiales*
MGS429	unclassified	*unclassified*
MGS231	Lachnospiraceae	*unclassified Lachnospiraceae*
MGS284	Prevotellaceae	*Prevotella*
MGS630	Prevotellaceae	*Prevotella*
MGS370	Prevotellaceae	*Prevotella*
MGS170	Prevotellaceae	*unclassified Prevotellaceae*
MGS344	Spirochaetaceae	*Treponema*
MGS382	unclassified	*unclassified*
MGS375	unclassified Clostridiales	*unclassified Clostridiales*
MGS015	Prevotellaceae	*Prevotella*
MGS152	unclassified Clostridiales	*unclassified Clostridiales*
MGS230	unclassified Bacteroidales	*unclassified Bacteroidales*
MGS059	Prevotellaceae	*Prevotella*
MGS606	unclassified Bacteroidetes	*unclassified Bacteroidetes*
MGS044	unclassified Clostridiales	*unclassified Clostridiales*
MGS264	Methanobacteriaceae	*Methanobrevibacter*
MGS217	unclassified Bacteroidales	*unclassified Bacteroidales*
MGS100	unclassified	*unclassified*
MGS029	unclassified Clostridiales	*unclassified Clostridiales*
MGS098	Prevotellaceae	*unclassified Prevotellaceae*

MGS135	Prevotellaceae	*Prevotella*
MGS009	unclassified Clostridiales	*unclassified Clostridiales*
MGS569	Prevotellaceae	*Prevotella*
MGS007	unclassified	*unclassified*
MGS035	unclassified	*unclassified*
MGS294	Prevotellaceae	*unclassified Prevotellaceae*
MGS522	Lachnospiraceae	*unclassified Lachnospiraceae*
MGS200	Succinivibrionaceae	*Ruminobacter*
MGS262	Prevotellaceae	*Prevotella*
MGS187	Prevotellaceae	*unclassified Prevotellaceae*
MGS373	unclassified	*unclassified*
MGS196	Fibrobacteraceae	*Fibrobacter*
MGS527	unclassified Clostridiales	*unclassified Clostridiales*
MGS583	Lachnospiraceae	*unclassified Lachnospiraceae*
MGS057	unclassified	*unclassified*
MGS087	unclassified Clostridiales	*unclassified Clostridiales*
MGS237	Acidaminococcaceae	*Succiniclasticum*
MGS290	Bifidobacteriaceae	*Bifidobacterium*
MGS188	Prevotellaceae	*Prevotella*
MGS361	unclassified Bacteroidales	*unclassified Bacteroidales*
MGS496	unclassified Bacteroidales	*unclassified Bacteroidales*
MGS164	unclassified	*unclassified*
MGS163	unclassified Bacteroidales	*unclassified Bacteroidales*
MGS338	Prevotellaceae	*Prevotella*
MGS083	Prevotellaceae	*Prevotella*
MGS074	Prevotellaceae	*Prevotella*
MGS154	Ruminococcaceae	*Ruminococcus*
MGS260	unclassified Bacteroidales	*unclassified Bacteroidales*
MGS125	Prevotellaceae	*Prevotella*
MGS039	unclassified	*unclassified*
MGS383	unclassified Bacteroidetes	*unclassified Bacteroidetes*
MGS075	Lachnospiraceae	*unclassified Lachnospiraceae*
MGS396	unclassified Thermoplasmata	*unclassified Thermoplasmata*
MGS281	Prevotellaceae	*Prevotella*
MGS315	Lachnospiraceae	*unclassified Lachnospiraceae*
MGS041	unclassified Clostridiales	*unclassified Clostridiales*
MGS483	Prevotellaceae	*Prevotella*
MGS477	unclassified	*unclassified*
MGS049	Prevotellaceae	*Prevotella*
MGS175	Prevotellaceae	*Prevotella*
MGS197	unclassified	*unclassified*
MGS599	unclassified	*unclassified*
MGS249	unclassified Bacteroidales	*unclassified Bacteroidales*
MGS019	Prevotellaceae	*Prevotella*

MGS137	unclassified	*unclassified*
MGS182	Lachnospiraceae	*unclassified Lachnospiraceae*
MGS063	unclassified	*unclassified*
MGS497	Clostridiaceae	*Clostridium*
MGS160	Prevotellaceae	*Prevotella*
MGS516	unclassified	*unclassified*
MGS244	Prevotellaceae	*Prevotella*
MGS094	Prevotellaceae	*Prevotella*
MGS519	unclassified Bacteroidales	*unclassified Bacteroidales*
MGS342	unclassified Clostridiales	*unclassified Clostridiales*
MGS251	unclassified	*unclassified*
MGS604	unclassified	*unclassified*
MGS116	Succinivibrionaceae	*unclassified Succinivibrionaceae*
MGS161	unclassified	*unclassified*
MGS241	unclassified Clostridiales	*unclassified Clostridiales*
MGS062	Prevotellaceae	*Prevotella*
MGS292	unclassified Bacteroidales	*unclassified Bacteroidales*
MGS103	Prevotellaceae	*Prevotella*
MGS172	unclassified	*unclassified*
MGS022	unclassified Clostridiales	*unclassified Clostridiales*
MGS178	Prevotellaceae	*Prevotella*
MGS140	Prevotellaceae	*Prevotella*
MGS535	unclassified	*unclassified*
MGS440	Ruminococcaceae	*Ruminococcus*
MGS055	unclassified	*unclassified*
MGS248	unclassified Bacteroidales	*unclassified Bacteroidales*
MGS530	Prevotellaceae	*Prevotella*
MGS407	unclassified Bacteroidales	*unclassified Bacteroidales*
MGS242	Prevotellaceae	*Prevotella*
MGS048	Lachnospiraceae	*unclassified Lachnospiraceae*
MGS053	Prevotellaceae	*Prevotella*
MGS205	Ruminococcaceae	*Ruminococcus*
MGS491	Prevotellaceae	*Prevotella*
MGS261	Prevotellaceae	*Prevotella*
MGS323	Prevotellaceae	*Prevotella*
MGS590	Clostridiaceae	*Clostridium*
MGS286	Prevotellaceae	*Prevotella*
MGS619	unclassified	*unclassified*
MGS316	Prevotellaceae	*Prevotella*
MGS313	Prevotellaceae	*Prevotella*
MGS151	unclassified Clostridiales	*unclassified Clostridiales*
MGS398	Prevotellaceae	*Prevotella*
MGS489	unclassified	*unclassified*
MGS105	unclassified Clostridiales	*unclassified Clostridiales*

MGS193	unclassified Bacteroidales	*unclassified Bacteroidales*
MGS245	unclassified Bacteroidales	*unclassified Bacteroidales*
MGS542	Spirochaetaceae	*Treponema*
MGS507	unclassified	*unclassified*
MGS307	Ruminococcaceae	*unclassified Ruminococcaceae*
MGS076	unclassified	*unclassified*
MGS379	Methanobacteriaceae	*Methanobrevibacter*
MGS145	unclassified Bacteroidales	*unclassified Bacteroidales*
MGS432	Ruminococcaceae	*Ruminococcus*
MGS303	unclassified Clostridiales	*unclassified Clostridiales*
MGS505	Prevotellaceae	*Prevotella*
MGS222	Prevotellaceae	*Prevotella*
MGS283	Ruminococcaceae	*Ruminococcus*
MGS362	Prevotellaceae	*Prevotella*
MGS110	unclassified	*unclassified*
MGS020	unclassified	*unclassified*
MGS149	unclassified Bacteroidales	*unclassified Bacteroidales*
MGS414	unclassified Bacteroidales	*unclassified Bacteroidales*
MGS372	Eubacteriaceae	*Eubacterium*
