# Supplementary material for: Identification of rumen microbial biomarkers linked to methane emission in Holstein dairy cows
Source: J Anim Breed Genet. 2019 Aug 16;137(1):49–59. doi: 10.1111/jbg.12427 (PMC6972549; doi:10.1111/jbg.12427)
Supplement: Supplementary file 7 [file JBG-137-49-s007.pdf]

Module	Description
M00046	Pyrimidine degradation, uracil => beta-alanine, thymine => 3-aminoisobutanoate
M00811	Nicotine degradation, pyrrolidine pathway, nicotine => succinate semialdehyde
M00104	Bile acid biosynthesis, cholesterol => cholate/chenodeoxycholate
M00173	Reductive citrate cycle
M00345	Formaldehyde assimilation
M00171	C4-dicarboxylic acid cycle, NAD - malic enzyme type
M00072	N-glycosylation by oligosaccharyltransferase
M00135	GABA biosynthesis, eukaryotes, putrescine
M00673	cephamycin C
M00169	Crassulacean acid metabolism
M00672	Penicillin biosynthesis
M00172	C4-dicarboxylic acid cycle, NADP - malic enzyme type
M00799	dTDP-L-oleandrose biosynthesis
M00039	Monolignol biosynthesis, phenylalanine/tyrosine => monolignol
M00798	TDP-L-mycarose biosynthesis
M00059	Glycosaminoglycan biosynthesis, heparan sulfate backbone
M00086	beta-Oxidation, acyl-CoA synthesis
M00098	Acylglycerol degradation
M00094	Ceramide biosynthesis
M00099	Sphingosine biosynthesis
M00374	Dicarboxylate-hydroxybutyrate cycle
M00567	Methanogenesis, CO2 => methane
M00014	Glucuronate pathway
M00168	Crassulacean acid metabolism
M00309	Non-phosphorylative Entner-Doudoroff pathway, gluconate/galactonate => glycerate
M00422	Acetyl-CoA pathway, CO2 => acetyl-CoA
M00531	Assimilatory nitrate reduction, nitrate => ammonia
M00113	Jasmonic acid biosynthesis
M00623	Phthalate degradation, phthalate => protocatechuate
M00001	Glycolysis (Embden-Meyerhof pathway), glucose => pyruvate
M00554	Nucleotide sugar biosynthesis, galactose => UDP-galactose
M00126	Tetrahydrofolate biosynthesis, GTP => THF
M00552	D-galactonate degradation D-galactonate => glycerate-3P
M00526	Lysine biosynthesis, DAP dehydrogenase pathway, aspartate => lysine
M00632	Galactose degradation, Leloir pathway, galactose => alpha-D-glucose-1P
M00120	Coenzyme A biosynthesis, pantothenate => CoA
M00176	Assimilatory sulfate reduction, sulfate => H2S
M00308	Semi-phosphorylative, gluconate => glycerate-3P
M00432	Leucine biosynthesis, 2-oxoisovalerate => 2-oxoisocaproate
M00115	NAD biosynthesis, aspartate => NAD
M00116	Menaquinone biosynthesis, chorismate => menaquinone
M00026	Histidine biosynthesis, PRPP => histidine

M00017	Methionine biosynthesis, apartate => homoserine => methionine
M00166	glyceraldehyde-3-phosphate dehydrogenase
M00015	Proline biosynthesis, glutamate => proline
M00096	C5 isoprenoid biosynthesis, non-mevalonate pathway.
M00071	Glycosphingolipid biosynthesis, neolacto-series, LacCer => nLc4Cer
M00535	Isoleucine biosynthesis, pyruvate => 2-oxobutanoate
M00023	Tryptophan biosynthesis, chorismate => tryptophan
M00040	Tyrosine biosynthesis, prephanate => pretyrosine => tyrosin
M00596	Dissimilatory sulfate reduction, sulfate
M00048	Inosine monophosphate biosynthesis, PRPP + glutamine
M00051	Uridine monophosphate biosynthesis, glutamine
M00002	Glycolysis, core module involving three-carbon compounds
M00003	Gluconeogenesis, oxaloacetate => fructose-6P. Definition,
