# Supplementary material for: Identification of rumen microbial biomarkers linked to methane emission in Holstein dairy cows
Source: J Anim Breed Genet. 2019 Aug 16;137(1):49–59. doi: 10.1111/jbg.12427 (PMC6972549; doi:10.1111/jbg.12427)
Supplement: Supplementary file 8 [file JBG-137-49-s008.pdf]

Identification of rumen microbial biomarkers linked to methane emission in Holstein dairy cows

Yuliaxis Ramayo-Caldas[1,2*], Laura Zingaretti[3], Milka Popova[4], Jordi Estellé[1], Aurelien Bernard[4], Nicolas Pons[5], Pau Bellot[3], Núria Mach[1], Andrea Rau[1], Hugo Roume[5], Miguel Perez-Enciso[3], Philippe Faverdin[6], Nadège Edouard[6], Dusko Ehrlich[5], Diego P. Morgavi[4], Gilles Renand[1*]

1UMR 1313 GABI, INRA, AgroParisTech, Université Paris-Saclay, Jouy-en-Josas, France ; 2Animal Breeding and Genetics Program, IRTA, Torre Marimon, Caldes de Montbui, 08140, Spain; 3Department of Animal Genetics, CRAG, UAB, Bellaterra, 08193, Spain. 4 Université Clermont Auvergne, INRA, VetAgro Sup, UMR 1213 Herbivores, F-63122 Saint-Genès-Champanelle, France; 5 INRA METAGENOPOLIS Unit Jouy-en-Josas, France; 6 UMR 1348 PEGASE, INRA, Agrocampus-Ouest, Saint-Gilles, France

[*]corresponding authors:

yuliaxis.ramayo@irta.cat

gilles.renand@inra.fr

Supplementary Material and Methods

Whole-metagenome sequencing and analysis

DNA was quantitated using Qubit Fluorometric Quantitation (ThermoFisher Scientific, Waltham, US) and qualified using DNA size profiling on a Fragment Analyzer (Agilent Technologies, Santa Clara, US). 3 µg of high molecular weight DNA (>10 kbp) was used to build the library. Shearing of DNA into fragments of approximately 150 bp was performed using an ultrasonicator (Covaris, Woburn, US) and DNA fragment library construction was performed using the Ion Xpress Plus gDNA Fragment Library Kit (ThermoFisher Scientific, Waltham, US). Purified and amplified DNA fragment libraries were sequenced using the Ion Proton Sequencer (ThermoFisher Scientific, Waltham, US), with a minimum of 20 million high-quality reads of 150 bp generated per library. After remove low quality and host contaminant reads, filtered high-quality reads were mapped with an identity threshold of 95% to the 16.6 million gene catalog using Bowtie2 included in the METEOR software (Cotillard et al. 2013). The gene abundance profiling table was generated by means of a two-step procedure using METEOR (Cotillard et al. 2013). First, the unique mapped reads (reads mapped to a unique gene in the catalogue) were attributed to their corresponding genes. Second, the shared reads (reads that mapped with the same alignment score to multiple genes in the catalogue) were attributed according to the ratio of the unique mapping counts of the corresponding genes. The gene abundance table was processed for normalization, and further analysis using the *MetaOMineR* R package (Prifti and Chatelier 2015). To reduce technical biases due to variable sequencing depth and avoid any artifacts of sample size on low abundance genes, read counts were rarefied. The gene abundance table was rarefied to 7 238 000 reads per sample by random sampling of mapped reads without replacement. The resulting rarefied gene abundance table was normalized according to the fragments per kilo base per million mapped reads (FPKM) strategy (normalization by the gene size and the number of total mapped reads reported in frequency) to obtain the gene abundance profile table. We subsequently identified metagenomic species (MGS) clusters, which are co-abundant gene groups with more than 500 genes corresponding to microbial species. Using the MSPMiner software (Cervino et al. 2018), 868 gene clusters (including 608 MGS with more than 500 genes) were clustered. MGS abundances were estimated as the mean abundance of the 50 genes defining a robust centroid of each cluster (if more than 10% of these genes gave positive signals). MGS taxonomic annotation was performed using all genes by sequence similarity using NCBI

blastn; a species-level name was assigned if >50% of the genes matched the same reference genome of the NCBI database (September 2017 release) at a threshold of 95% identity and 90% gene length coverage. The remaining MGS were assigned to a given taxonomic level, from genus to superkingdom level, if more than 50% of their genes had the same level of assignment. Microbial gene richness (gene count) was calculated by counting the number of genes that were detected at least once in a given sample. MGS richness (MGS count) was calculated directly from the MGS abundance matrix.

To assess the metabolic potential of the rumen microbiome, each gene of the catalog was annotated to the KEGG orthologous genes database (KEGG [Kyoto Encyclopedia of Genes and Genomes] release 8.2) (Kanehisa and Goto 2000) using the diamond software (Buchfink et al. 2014) (bitscore > 60). The relative abundance of each KEGG orthologous (KO) group was estimated as the sum of the abundance of genes annotated to the same KO. In addition, the relative abundance of each KEGG module was estimated in a decompartmentalized way using the reaction pathway (and alternative pathway) structure definition. A KEGG module was reported if at least 90% of its reactions were covered by KO detected in a sample. Relative abundance of reported modules was estimated as the mean abundance of each reaction (KO) in a pathway. Non-metric multidimensional scaling (nMDS) analyses were done with *vegan* (Jari Oksanen et al. 2018) using the Bray-Curtis and binary Jaccard dissimilarities estimated from the shotgun gene count matrix. PERMANOVA tests were performed with the *adonis* function in *vegan* (Jari Oksanen et al. 2018) and the Bray-Curtis and binary Jaccard matrices.

Supplementary Results

Composition of the rumen bacterial and archaeal communities

In agreement with previous studies (Huws et al. 2018; Li et al. 2018; Wallace et al. 2015), the most abundant bacterial phyla were Bacteroidetes (56.5%), Firmicutes (26.1%) and Fibrobacteres (8.3%). Likewise, predominant families were Prevotellaceae (46.2%), Rumminococcaeae (8.3%) and Fibrobacteraceae (6.3%). Regarding archaea, the ruminal community was dominated by members of the Methanobrevibacter (*gottschalkii* and *ruminantium* clades) and Methanosphaera genera. To provide an estimate of the within-individual variability of the ruminal bacteria community, repeated

measures (two successive months) were used; the RV-coefficient was R=0.64, suggesting moderate repeatability between time points. Furthermore, no significant differences were observed for diversity index levels between time points (**Supplementary Figure 1**).

Supplementary tables:

Supplementary Table 1. Cow diet composition throughout the experiment

Supplementary table 2. Description of the differentially-abundant bacterial genera detected between ruminotype clusters.

Supplementary table 3. Description of the differentially-abundant archaeal OTUs detected between ruminotype clusters.

Supplementary table 4. Description of OTUs detected by the multivariate analysis.

Supplementary table 5. Description of MGS detected in the sPLS-DA analysis based on ruminotype clusters classification of cows.

Supplementary table 6. Description of the differentially-abundant MGS detected between ruminotype clusters.

Supplementary table 7. Description of KEGG modules detected in the sPLS-DA analysis based on ruminotype clusters classification of cows.

Supplementary figures:

Supplementary Figure 1. Comparison of diversity indices between period one (red) and two (blue) for the *n*=21 cows in batch B1.

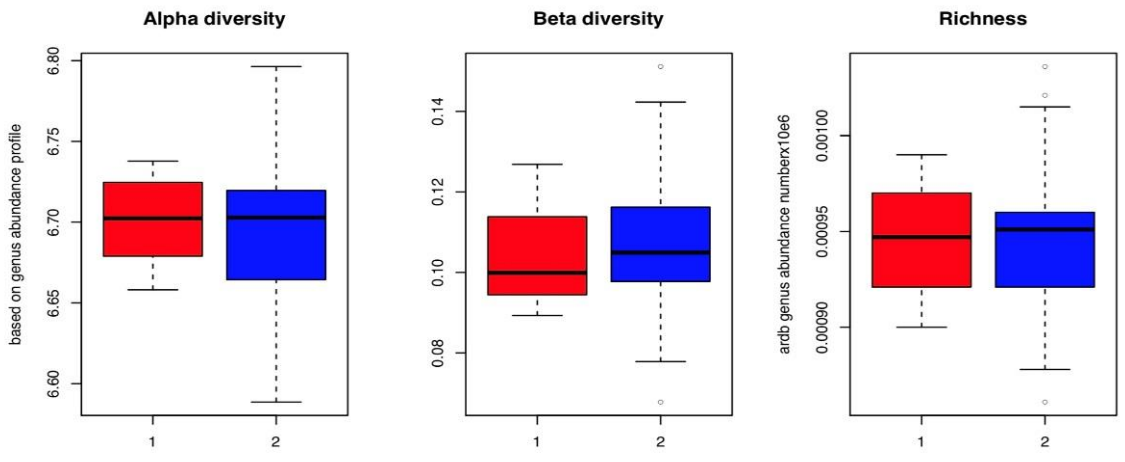

Supplementary Figure 2. (A) OTU sample distribution. (B) Area under the ROC curve corresponding to model ability to correctly classify samples.

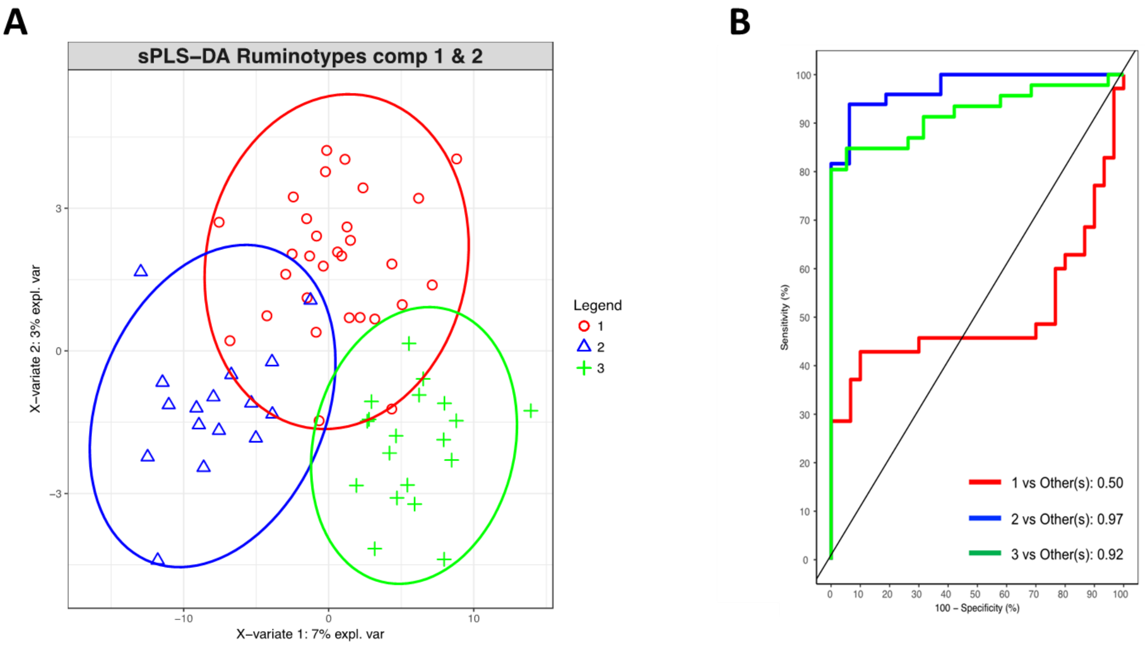
A
sPLS–DA Ruminotypes comp 1 & 2
X–variate 1: 7% expl. var
X–variate 2: 3% expl. var
Legend
1
2
3
B
Sensitivity (%)
100 – Specificity (%)
1 vs Other(s): 0.50
2 vs Other(s): 0.97
3 vs Other(s): 0.92

Supplementary Figure 3. Non-metric multidimensional scaling analyses based on whole-metagenome gene abundances and their relation with ruminotypes (left) and CH4y phenotype (rigth). (A) and (B) Bray-Curtis dissimilarity nMDS; (C) and (D) Binary Jaccard dissimilarity nMDS

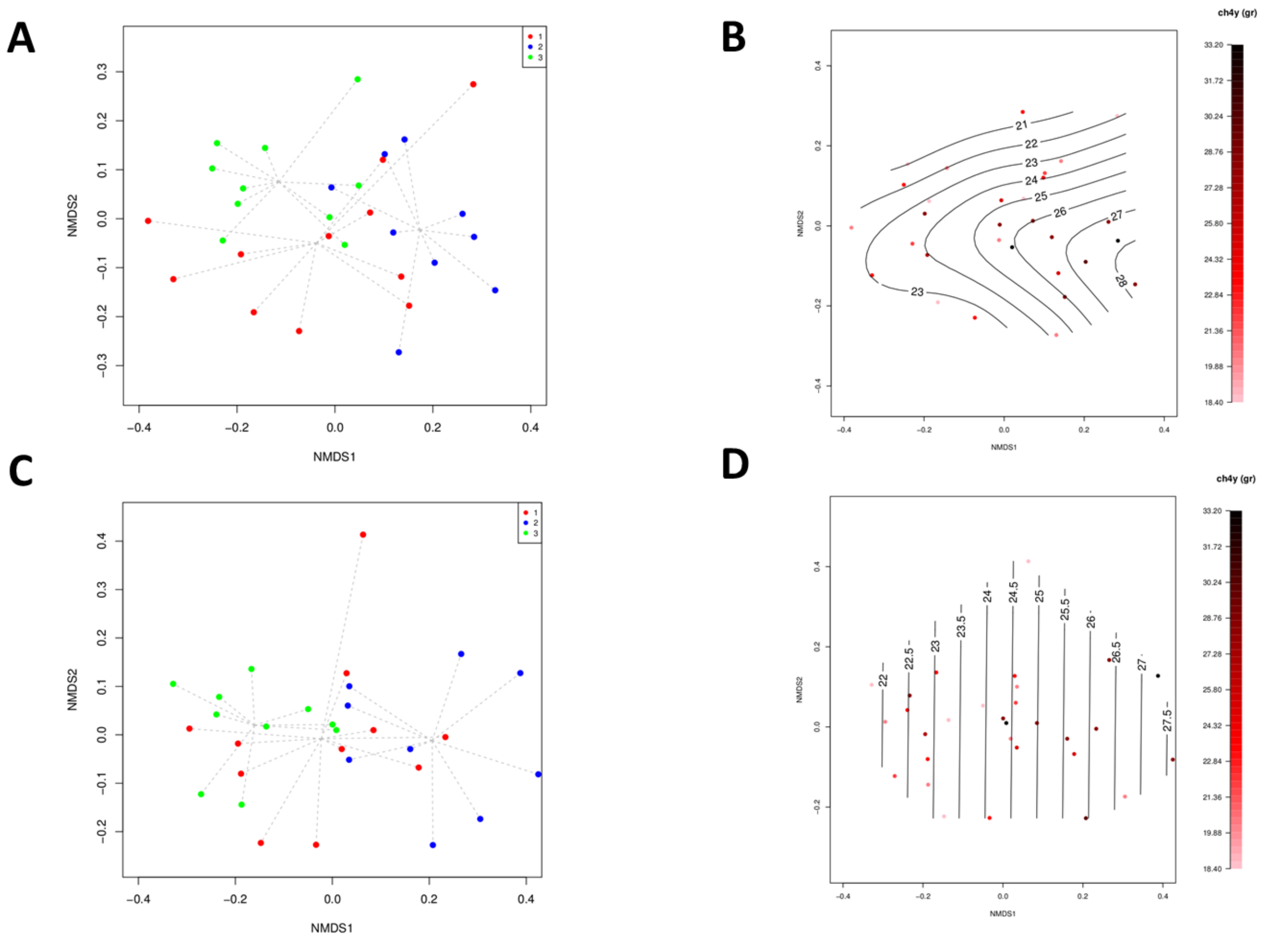

Supplementary Figure 4. (A) MGS sample distribution. (B) Area under the ROC curve corresponding to model ability to correctly classify samples.

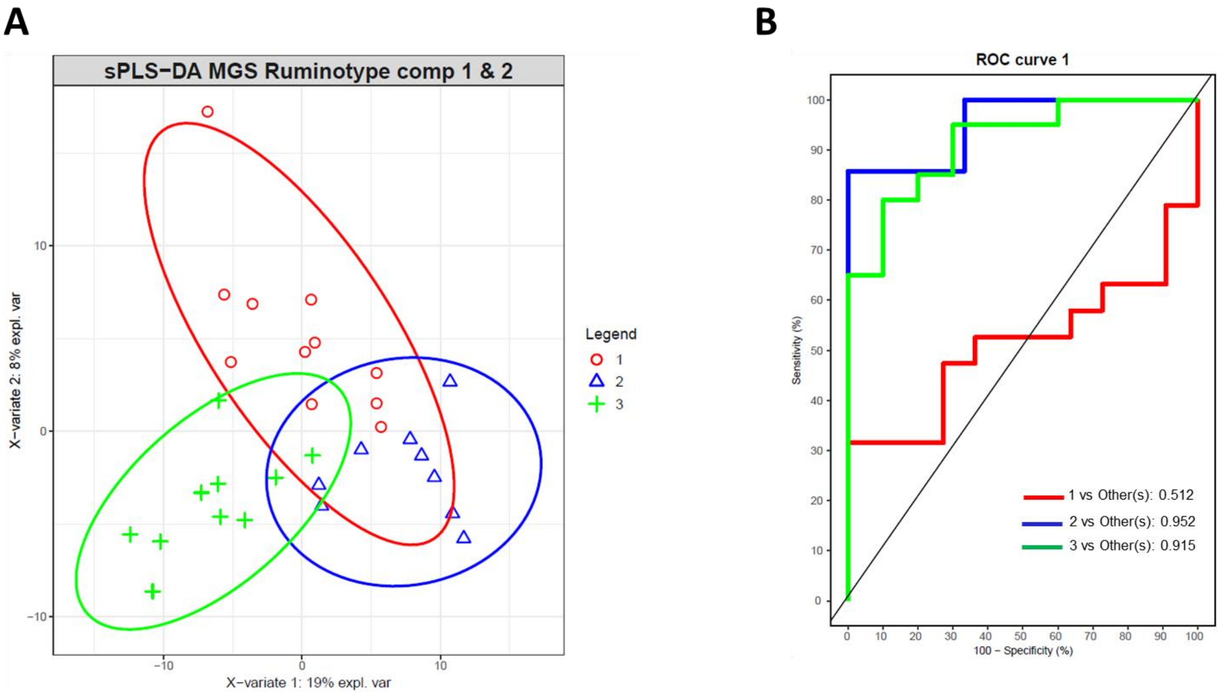

Supplementary Figure 5. (A) KEEG module sample distribution. (B) Area under the ROC curve corresponding to model ability to correctly classify samples.

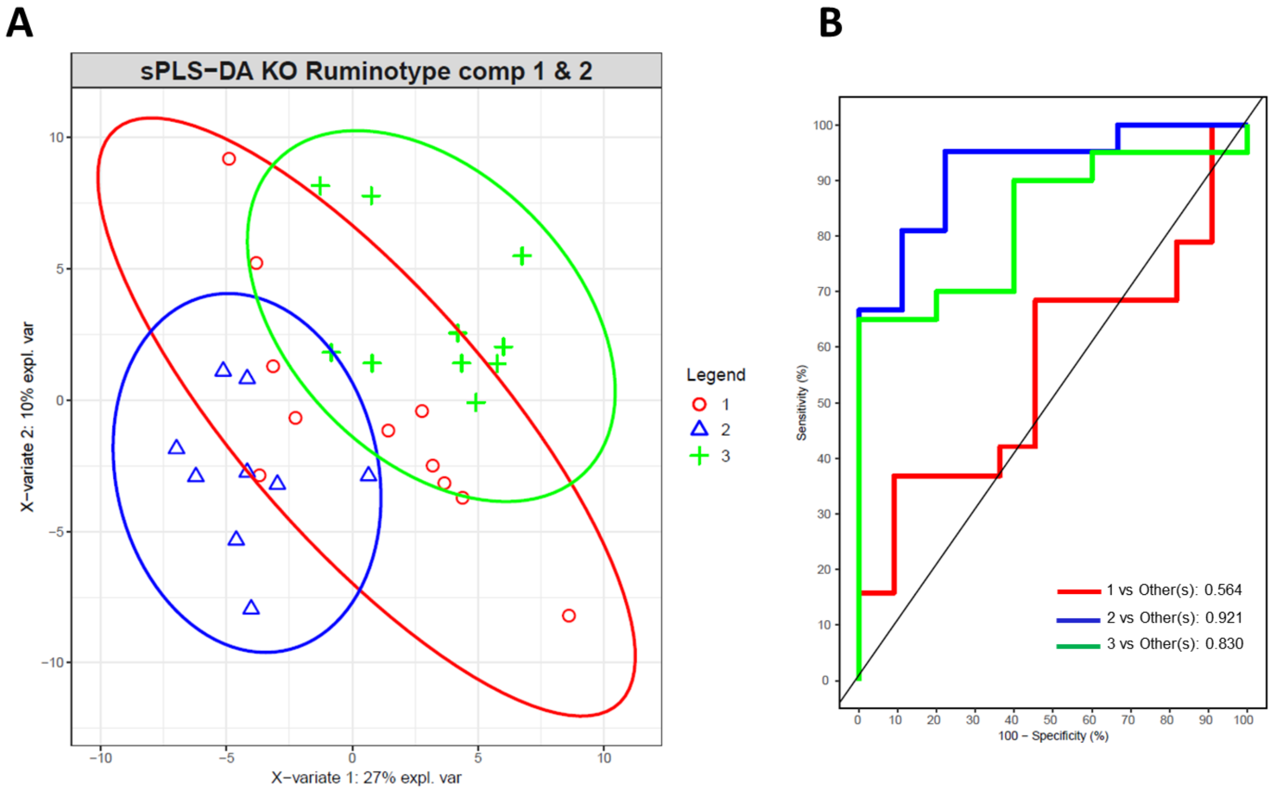

Supplementary Figure 6. Mean description of the differentially-abundances KOs from Methanogenesis pathway.

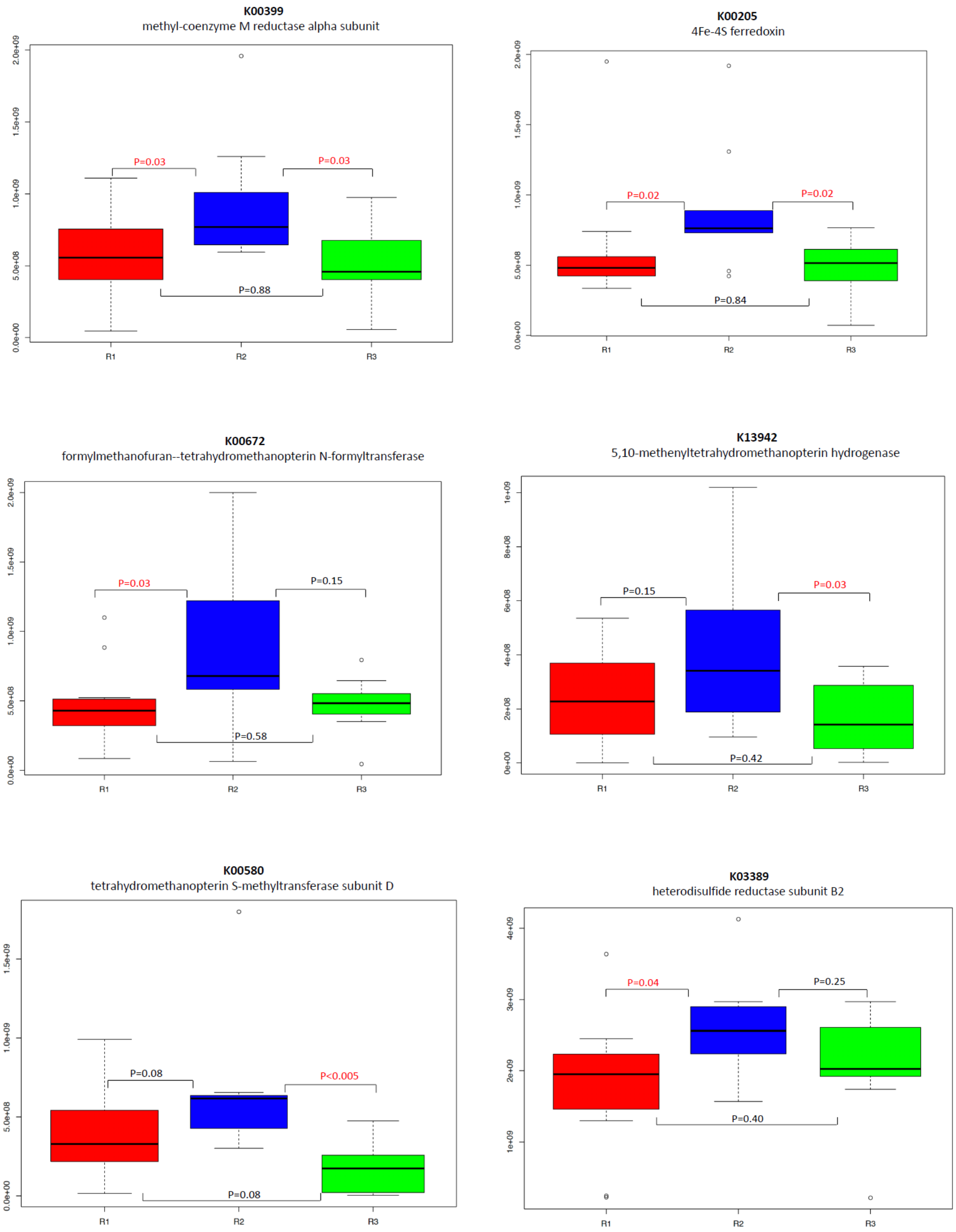

Reference

Buchfink B, Xie C, and Huson DH. 2014. Fast and sensitive protein alignment using DIAMOND. Nature Methods 12:59.

Cervino ACL, Pichaud M, Plaza Oñate F, Le Chatelier E, Gauthier F, Almeida M, Ehrlich SD, and Magoulès F. 2018. MSPminer: abundance-based reconstitution of microbial pan-genomes from shotgun metagenomic data.

Cotillard A, Kennedy SP, Kong LC, Prifti E, Pons N, Le Chatelier E, Almeida M, Quinquis B, Levenez F, Galleron N et al. . 2013. Dietary intervention impact on gut microbial gene richness. Nature 500:585.

Huws SA, Creevey CJ, Oyama LB, Mizrahi I, Denman SE, Popova M, Muñoz-Tamayo R, Forano E, Waters SM, Hess M et al. . 2018. Addressing Global Ruminant Agricultural Challenges Through Understanding the Rumen Microbiome: Past, Present, and Future. Frontiers in Microbiology 9:2161.

Jari Oksanen F, Blanchet G, Friendly M, Kindt R, Legendre P, McGlinn D, Minchin PR, O'Hara RB, Simpson GL, Solymos P et al. . 2018. vegan: Community Ecology Package. R package version 2.5-3. https://CRANR-projectorg/package=vegan.

Kanehisa M, and Goto S. 2000. KEGG: Kyoto Encyclopedia of Genes and Genomes. Nucleic Acids Research 28(1):27-30.

Li J, Zhong H, Ramayo-Caldas Y, Terrapon N, Lombard V, Potocki-Veronese G, Estelle-Fabrellas J, Popova M, Yang Z, Zhang H et al. . 2018. A catalog of microbial genes from the bovine rumen reveals the determinants of herbivory. bioRxiv.

Prifti E, and Chatelier EL. 2015. Mining Metaomics Data (MetaOMineR). https://cran.r-project.org/web/packages/momr/index.html. p 'MetaOMineR' suite is a set of R packages that offers many functions and modules needed for the analyses of quantitative metagenomics data. 'momr' is the core package and contains routines for biomarker identification and exploration. Developed since the beginning of field, 'momr' has evolved and is structured around the different modules such as preprocessing, analysis, vizualisation, etc.

Wallace RJ, Rooke JA, McKain N, Duthie C-A, Hyslop JJ, Ross DW, Waterhouse A, Watson M, and Roehe R. 2015. The rumen microbial metagenome associated with high methane production in cattle. BMC Genomics 16(1):839.
